# Kinetics of rapamycin production by *Streptomyces hygroscopicus* MTCC 4003

**DOI:** 10.1007/s13205-013-0189-2

**Published:** 2013-12-01

**Authors:** Subhasish Dutta, Bikram Basak, Biswanath Bhunia, Samayita Chakraborty, Apurba Dey

**Affiliations:** 1 Department of Biotechnology, National Institute of Technology Durgapur, Mahatma Gandhi Avenue, Durgapur, 713209 India; 2 Department of Bio Engineering, National Institute of Technology Agartala, Barjala, Tripura 799055 India

**Keywords:** Rapamycin, Growth kinetics, *Streptomyces hygroscopicus*, Antibiotic, Substrate inhibition

## Abstract

Research work was carried out to describe the kinetics of cell growth, substrate consumption and product formation in batch fermentation of rapamycin using shake flask as well as laboratory-scale fermentor. Fructose was used as the sole carbon source in the fermentation media. Optimization of fermentation parameters and reliable mathematical models were used for the maximum production of rapamycin from *Streptomyces hygroscopicus* MTCC 4003. The experimental data for microbial production of rapamycin fitted well with the proposed mathematical models. Kinetic parameters were evaluated using best fit unstructured models, viz. Andrew’s model, Monod model, Yano model, Aiba model. Andrew’s model showed a comparatively better *R*^2^ value (0.9849) among all tested models. The values of maximum specific growth rate (*μ*_max_), saturation constant (*K*_S_), inhibition constant (*K*_i_), and growth yield coefficient (*Y*_X/S_) were found to be 0.008 (h^−1^), 2.835 (g/L), 0.0738 (g/L), and 0.1708 (g g^−1)^, respectively. The optimum production of rapamycin was obtained at 300 rpm agitation and 1 vvm aeration rate in the fermentor. The final production of rapamycin in shake flask was 539 mg/L. Rapamycin titer found in bioreactor was 1,316 mg/L which is 52 % higher than the latest maximum value reported in the literature.

## Introduction

Rapamycin, also known as sirolimus, is an antibiotic commonly used as a potent antifungal and immunosuppressant drug produced by the soilborne actinomycete *Streptomyces hygroscopicus* (Fang and Demain 1995). It has also been reported to have antitumor, neuroprotective and anti-aging properties (Zou and Li 2013). In recent time, because of its exceptional biological and pharmaceutical potential it has generated interest for its therapeutic use (Graziani 2009; Sehgal 2003). Its immunosuppressive activity due to inhibition of T-cell activation and proliferation has led to its potential use in clinical treatment of graft rejection in organ transplant (Weber et al. 2005) and autoimmune diseases such as rheumatoid arthritis (Foroncewicz et al. 2005). In addition to its therapeutic usefulness, semisynthetic derivatives of rapamycin has also been shown to display activities against cancer (Park et al. 2010), Parkinson’s disease (Tain et al. 2009), and AIDS (Nicoletti et al. 2009). Its anti-aging activity has been published recently when scientists observed its ability to extend the life span of mice (Zou and Li 2013).

The mechanism of action of rapamycin is distinct from that of cyclosporine A and FK-506. The latter drugs inhibit the first phase of T-cell activation by blocking calcineurin, serine/threonine phosphatase (transcriptional activator of IL-2 gene); whereas, rapamycin interferes with the second phase of T-cell activation by blocking the IL-2 dependent signal transduction (Morelon et al. 2001). The anti-proliferative effects of rapamycin are mediated through the formation of an active complex with a cytosolic protein FK-506 and binding protein (FKBP12) allowing this drug receptor complex to interact with a putative lipid kinase (Wiederrecht et al. 1995), and inhibits the 289 kDa RAFT/FRAP proteins called mTOR (mammalian target of rapamycin) (Sabatini et al. 1995).

Despite the versatile activities and resultant demand of this drug, its use may be trimmed because of the low titer of rapamycin produced by *S. hygroscopicus*. The low titer of this drug produced by the organism has now become a rate-limiting factor in further development and industrialization of this natural product (Zhu et al. 2010). In the past decades, most efforts have focussed on rapamycin biosynthesis (Park et al. 2010; Graziani 2009), its pharmaceutical activities (Prapagdee et al. 2008; Park et al. 2010; Weber et al. 2005; Foroncewicz et al. 2005; Nicoletti et al. 2009), strain improvement (Zhu et al. 2010; Xu et al. 2005; Chen et al. 2009) and optimization of physiological factors for better production of rapamycin (Zou and Li 2013; Chen et al. 2008). In the literature, although there are many reports describing optimization of the media (Refaat and Abdel-Fatah 2008) and improvement of strain for better production of rapamycin (Xu et al. 2005; Jung et al. 2011), kinetic studies of growth and rapamycin production by *S. hygroscopicus* have not been satisfactorily done yet. Though an attempt was made by Schuhmann and Bergter investigating the branch formation, and cytological properties of mycelial growth of *Streptomyces hygroscopicus* on solid media (Schuhmann and Bergter 1976), the actual growth and rapamycin production kinetic parameters were not determined.

The metabolism and product formation pattern of a microorganism depend mainly on their fermentative, nutritional, physiological, and genetic nature (Prakasham et al. 2007; Bhunia et al. 2012). Exploitation of such microbial metabolism for the desired product formation by regulating the critical fermentation parameters helps in commercialization (Subba Rao et al. 2008). Hence, careful kinetic studies are required to monitor the growth of microorganisms and product formation pattern in presence of various substrates. Kinetic studies provide good quantitative information regarding the behavior of a system, which is essential for study of growth of the organism and consequent product formation (Bhunia et al. 2012).

In the present study, we used various unstructured kinetic models to characterize the growth and rapamycin production by *S. hygroscopicus*. The kinetic parameters obtained by fitting the experimental data of growth and product formation with the unstructured models can be effectively used to explain the relationship between microbial growth and substrate utilization.

## Materials and methods

### Chemicals

All chemicals used were of analytical and HPLC grade and purchased from Sigma Aldrich (USA), Himedia (India), and Merck (India). HPLC-grade rapamycin standard was purchased from Merck, Germany. Deionized water used for HPLC analysis was prepared by Ultrapure Water System (Arium^®^, 611UF, Sartorius, Germany).

### Microorganisms

*Streptomyces hygroscopicus* MTCC 4003 and *Candida albicans* MTCC 227 (test organism) were procured in lyophilized form from microbial type culture collection (MTCC) Chandigarh, India. *S. hygroscopicus* was grown and maintained in a medium consisting of (in g/L), glucose 4; yeast extract 4; malt extract 10; CaCO_3_ 2. The pH of the medium was maintained at 7.2. *C. albicans* was grown on MYGP medium having the following composition (g/L): malt extract 3; yeast extract 3; peptone 5; glucose 10 (pH 7).

### Preparation of inoculum

Inoculum (seed culture) was prepared by inoculating thawed *S. hygroscopicus* spores into 250-mL Erlenmeyer flask containing 100 mL growth medium with the help of a sterile inoculating loop under aseptic condition. The flasks were then incubated at 25 °C and 120 rpm for 7 days. *S. hygroscopicus* and *C. albicans* were maintained by bimonthly transfer to fresh medium and stored at 4 °C after incubation at 25 °C for 5 days.

### Fermentation in shake flask

Two percent (v/v) seed culture was transferred into the production media (previously optimized in our lab) having the following composition (g/L): fructose 22; mannose 5; malt extract 10; casein 0.3; (NH_4_)_2_SO_4_ 5.3; NaCl 5; K_2_HPO_4_ 4; ZnSO_4_·7H_2_O 0.06; MgSO_4_·7H_2_O 0.0025; MnSO_4_·H_2_O 0.012; FeSO_4_·7H_2_O 0.1; CoCl_2_·6H_2_0 0.010; Na_2_SO_4_ 0.3; CaCO_3_ 3 (pH 7.2). In another experiment, seed culture was inoculated into the same production media devoid of mannose where the amount of mannose was supplemented by fructose, as both have same empirical formula and molecular mass. Similarly, another two sets of production media were used which incorporated same amount of glucose, replacing fructose and mannose. Fructose, glucose and mannose were autoclaved separately and added to the production media aseptically so as to achieve the desired concentration in the media. Fermentation was carried out in 250-mL Erlenmeyer flasks, each contained 50 mL production medium and was incubated at 25 °C and 120 rpm for 7 days. All the experiments were performed in triplicate.

### Fermentation in stirred tank reactor (STR)

Further work was done in the 2.2-L stirred tank reactor (STR) with 2-L working volume and Biocommand Plus fermentation supervising software (New Brunswick Scientific Co. Inc. USA) for advanced online control which result in higher cell density and rapamycin productivity. The reactor vessel containing production medium was sterilized by autoclaving along with the silicon tubes of 0.5 cm diameter and reagent bottles containing NaOH, HCl, antifoam agent (silicon oil) and one empty reagent bottle used for transferring the inoculum at 15 psi and 121.5 °C for 15 min. The pH probe was calibrated before autoclaving the reactor and set to 7.24 while the dissolved oxygen (DO) probe was calibrated after autoclaving and set to 100 %. pH was maintained at the set point 7.24 by supplying 0.1 (N) NaOH and 0.1 (N) HCl automatically to the fermentor with the help of peristaltic pumps. DO level was controlled by agitator speed and aeration rate during fermentation. Agitation was set at 300 rpm with six-bladed turbine impellers. Compressed sterile air was sparged at 1 vvm cultivating for 7 days at 25 °C.

### Analytical procedures

During fermentation in shake flasks, the microbial growth under the submerged conditions appeared as spherical pellets. Hence, after inoculation, 50 mL of samples were taken from shake flask in 50 mL centrifuge tube at every 24 h interval starting from 0th h and centrifuged at 3,500 rpm for 15 min (Cheng 1995). The sample from STR was collected in every 24 h interval in a 50-mL centrifuge tube and centrifuged. After centrifugation, the supernatant obtained was taken in separate centrifuge tube and the cell pellet was washed twice with 3 mL of methanol by centrifuging at 200 rpm for 15 min. The methanolic extract was mixed with the supernatant and used for rapamycin concentration (Sallam et al. 2010). The cell pellets were dried at 80 °C to a constant weight for 48 h in a hot air oven. The biomass was expressed as dry weight in g/L.

Residual fructose concentration was determined by 3,5-dinitrosalicylic acid (DNS) method (Miller 1959). A standard calibration curve of optical density versus known concentration of fructose was prepared and the unknown concentration of residual fructose in the media was determined from this curve.

Bioassay determination of rapamycin was performed by “paper-disc agar diffusion method” as described by Kojima et al. (1995). The assay was conducted in agar plates of assay medium (“Microorganisms”) seeded with *C. albicans* MTCC 227 as test organism. From the mean diameter of the inhibition zones the concentration of rapamycin was empirically determined. Rapamycin concentration was determined using high performance liquid chromatography (HPLC). A calibration curve of different concentrations of rapamycin standard versus area of HPLC peak was plotted and unknown concentration of rapamycin in the supernatant was calculated using the linear equation obtained from the calibration curve. All experiments were done in triplicate. For sample preparation, the supernatant was filtered through 0.22-μm membrane filters for organic solvents (Pall Corporation, India). After appropriate dilutions with HPLC-grade methanol, samples were analyzed by HPLC system (Waters™ 600) equipped with UV/visible detector and a C_18_ hypersil column (4.6 × 250 mm; 5 μm particle size; Waters, USA). Mobile phase used was methanol:acetonitrile (80:20 v/v), at a flow rate of 1 mL/min. An aliquot of 20 μL of filtrate supernatant was injected and analyzed at 272 nm using the UV/visible detector.

### Mathematical background

In Monod’s model, the growth rate is related to the concentration of a single growth-limiting substrate through the parameters *μ*_max_ and *K*_S_. Monod model also relates the yield coefficient (*Y*_X/S_) to the *μ* (Okpokwasili and Nweke 2005). The specific growth rate in the exponential phase was calculated using the following equation:

1 $$\frac{\text{d}X}{\text{d}t}=\mu X$$

GraphPad Prism 5 software was used to calculate the kinetics parameters from the Monod equation:

2 $$\mu =\frac{\mu_{\text{max}}S}{K_{\text{S}}+S}$$

When higher substrate concentration inhibits cell growth, the original Monod model becomes unsatisfactory. In this case, Monod derivatives that provided corrections for substrate inhibition (by incorporating the inhibition coefficient *K*_i_) can be used to describe the kinetics study. Among the substrate inhibition models, the Andrew’s equation is most widely used (Okpokwasili and Nweke 2005). After evaluating several kinetic models (Yano, Aiba) the Andrew’s model was best fitted and gave the highest *R*^2^ value.

3 $$\mu =\frac{\mu_{\text{max}}S}{S^{2}K_{\text{i}}+S+K_{\text{S}}}$$

The substrate utilization kinetics is given by Eq. (4). A carbon substrate is used to form cell material and metabolic products as well as for maintenance of the cell.

4 $$\frac{\text{d}S}{\text{d}t}=-\frac{1}{Y_{\text{X/S}}}\frac{\text{d}X}{\text{d}t}-\frac{1}{Y_{\text{P/S}}}\frac{\text{d}P}{\text{d}t}-mX$$

However, if the substrate used for product formation and cell maintenance is assumed to be negligible, Eq (4) can be written as:

5 $$\frac{\text{d}S}{\text{d}t}=-\frac{1}{Y_{\text{X/S}}}\frac{\text{d}X}{\text{d}t}$$

Now, *Y*_X/S_ is the ratio of cell mass growth and substrate concentration used for cell growth. *Y*_X/S_ can be expressed as:

6 $$Y_{\text{X/S}}=-\frac{\text{d}X}{\text{d}S}$$

*Y*_X/S_ was calculated from experimental data using the Eq. (7).

7 $$Y_{\text{X/S}}=\frac{X-X_{0}}{S_{0}-S}$$

## Results and discussion

**Fig. 1:**
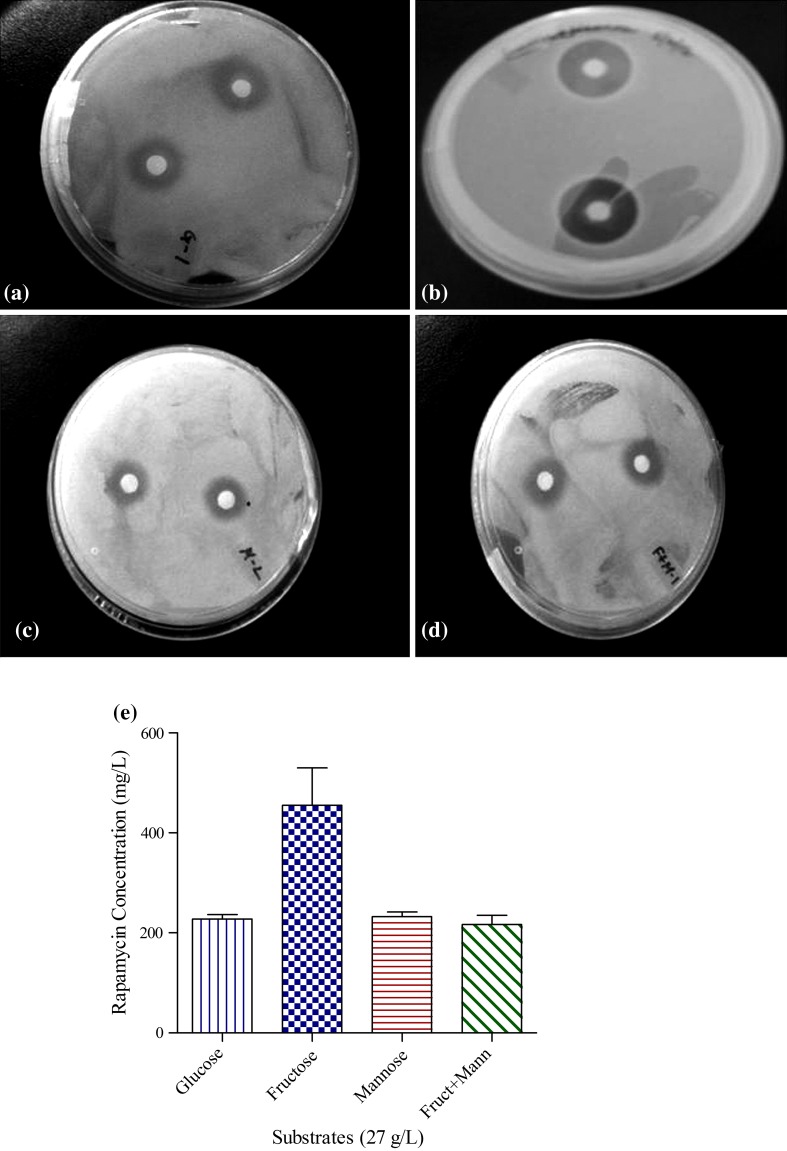
Inhibition zones of rapamycin on agar plates of *Candida albicans* obtained using different carbon sources: **a** glucose, **b** fructose, **c** mannose, **d** fructose + mannose. **e** Concentrations of rapamycin found in shake flask using above-mentioned carbon sources

For rapamycin production, we evaluated four different combinations (1. glucose, 2. fructose, 3. fructose + mannose and 4. mannose) of carbon sources for better production. It can be seen in Fig. 1, among the carbohydrates used fructose yielded highest rapamycin titer. Catabolite repression may be the most likely reason for this lagging effect (Priest 1977; Kumar et al. 1999). It was previously established that a catabolite control protein (CcpA) was responsible for this regulatory mechanisms which transduced signal for the repression in rapamycin. Synthesis (Tobisch et al. 1999). The result is as accordance with Kojima et al. (1995).Therefore, we selected fructose in further studies replacing all other carbon sources by the same amount as they have same molecular mass (180.16 g/mol) as well as same empirical formula (C_6_H_12_O_6_). Although carbohydrates are also present in other media constituents, viz. malt extract, it is hard to analyze the amount of sugar present in the constituents and the amount utilized. Hence, analysis of substrate utilization was done by assuming supplemented carbohydrates as sole source of carbon and energy, thereby neglecting the amount of carbohydrate present in other media constituents. This result contradicts the findings reported by Lee et al., where they showed that a combination of fructose and mannose resulted in good growth of the organisms and consequent higher rapamycin titer (Lee et al. 1997). However, our result suggested that fructose together with nitrogen source casein and (NH_4_)_2_SO_4_ supported good growth and rapamycin production by the employed strain (Fig. 1).

**Fig. 2:**
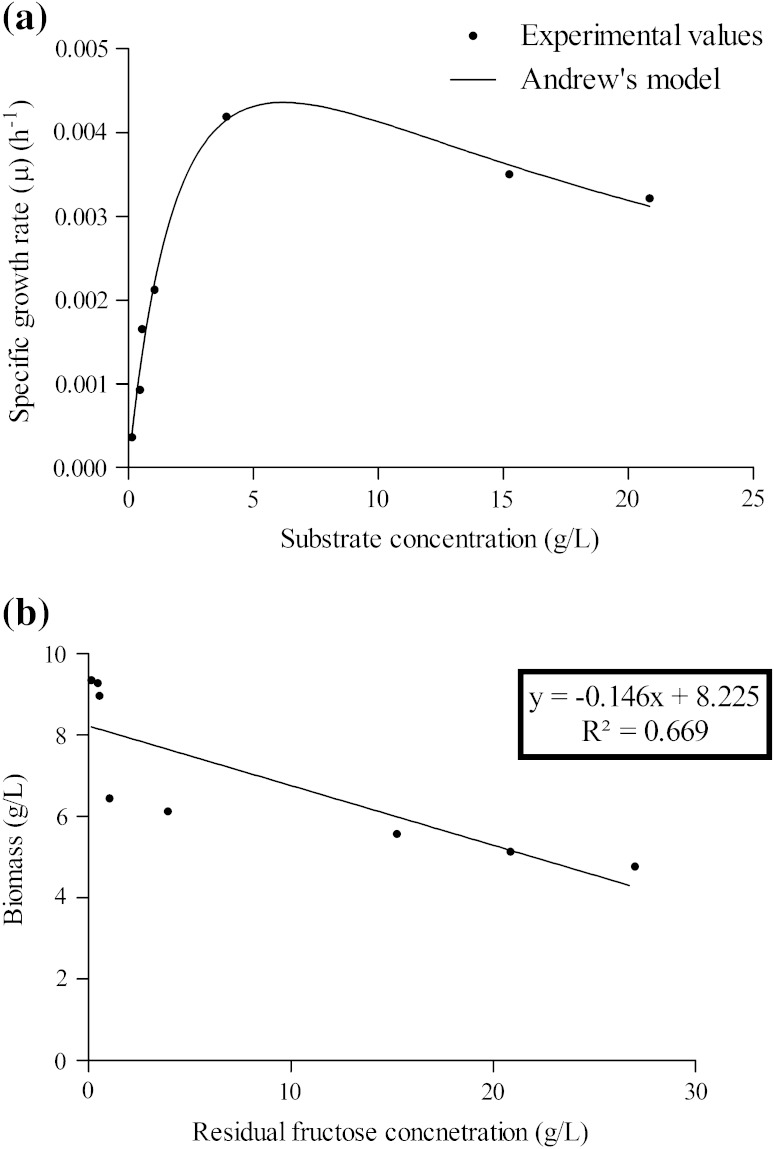
**a** Relationship between specific growth rate (*μ*) and substrate concentrations (*S*). **b** Determination of growth yield coefficient (*Y*_X/S_)

To study the effect of *S* on *μ*, *μ* for different *S* was calculated using Eq. (1) while *X* was obtained at different intervals of the incubation period. The values of *μ* for different *S* were fitted to Monod’s model using GraphPad Prism 5 software. The growth kinetic parameters, viz. *μ*_max_ and *K*_S_ were found to be 0.003869 h^−1^ and 0.8271 g/L. The lower correlation coefficient (*R*^2^) value of 0.8713 found in Monod’s model suggested that there was reasonable substrate or product inhibition on growth of the organism (Bhunia et al. 2012). Analysis of the Monod’s model under different substrate concentration conditions suggested that fructose concentration regulates the microbial growth pattern. Since, there was no report of product inhibition for rapamycin production, the effect of substrate inhibition was only considered for modeling. Several substrate inhibition kinetic models were examined and compared in this work (Table 1). Andrew’s model for substrate inhibition on microbial growth was found to fit the experimental values well, since an *R*^2^ value of 0.9849 was obtained (Fig. 2a). The values of *μ*_max_, *K*_S_ and *K*_i_ were found to be 0.0083 h^−1^, 2.835 and 0.073 g/L, respectively. The higher *R*^2^ value found with Andrew’s model indicates that it is comparatively better fitted model for the experimental data than Monod and other substrate inhibition models (Table 1). In rapamycin production, the increase in biomass concentration was accompanied by a decrease of fructose concentration. It is assumed that fructose is consumed for cell growth and cell maintenance. *Y*_X/S_ value was determined by averaging *Y*_X/S_ values calculated using Eq. (7) at different data points (Fig. 2b). Its values ranged from 0.0598 to 0.1708 and were found to be maximal at lower *S* and minimal at higher *S* because of the substrate inhibition on *Y*_X/S_. The average *Y*_X/S_ value was calculated to be 0.107 g g^−1^ and was fairly constant up to fructose concentration of 0.548 g/L and then decreased to minimal at fructose concentration of 27 g/L.

**Table 1:** Growth kinetic parameters for *S. hygroscopicus* MTCC 4003 obtained by different models

Mathematical models	*μ*_max_ (h^−1^)	*K*_S_ (g/L)	*K*_i_ (g/L)	*R* ^2^
Andrew’s model $\mu =\frac{\mu_{\text{max}}S}{S^{2}K_{\text{i}}+S+K_{\text{S}}}$	0.0083	2.835	0.073	0.9849
Yano model $\mu =\frac{\mu_{\text{max}}S}{S+K_{\text{S}}+\left(\frac{S^{2}}{K_{\text{i}}}+\frac{S^{3}}{K_{\text{i}}^{2}}\right)}$	0.0071	2.324	29.21	0.9818
Aiba model $\mu =\frac{\mu_{\text{max}}S}{K_{\text{S}}+S}e^{\left(-S+K_{\text{i}}\right)}$	0.0078	2.540	0.03963	0.9813
Monod model $\mu =\frac{\mu_{\text{max}}S}{K_{\text{S}}+S}$	0.003869	0.8271	–	0.8713

The fermentor was operated with constant impeller speed of 300 rpm for 8 consecutive days. The dissolved oxygen (DO) plays a vital role in the rapamycin production. The air flow rate was kept constant at 1 vvm throughout the fermentation process. Initially DO was set at 100 % at the time of inoculation, while during the lag phase microorganisms did not start to utilize the carbon of the media. However, when the fermentation enters the log phase, dissolved oxygen rapidly decreases as a result of increase in oxygen demand by the cells. Therefore, the dissolved oxygen level decreases and finally reached a certain stationary value. Finally, the DO level was maintained at 10 % of saturation level all the time (Zhu et al. 2010).

**Fig. 3:**
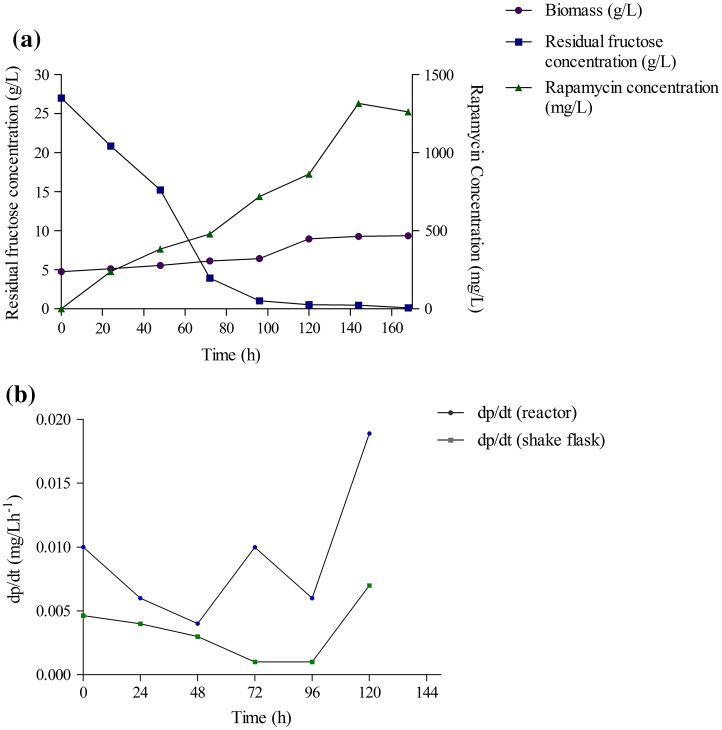
Time-course profile of cell growth, rapamycin production, and substrate utilization

An antibiotic is a secondary metabolite which is synthesized mainly at the stationary phase of growth of a microorganism. However, the obtained data clearly show that exponential phase of growth is very important for rapamycin production (Zhu et al. 2010; Sanchez and Brana 1996). *Streptomyces hygroscopicus* MTCC 4003 showed a conventional growth pattern during the batch fermentation in the bioreactor (Fig. 3a). The production of rapamycin was started after 24 h of incubation and reached a maximal at 144 h (Fig. 3a). Similar trends were also reported in the literature (Xu et al. 2005; Lee et al. 1997). Rapamycin production was observed to increase rapidly during end of the exponential phase and early stationary phase (Xu et al. 2005). The productivity (mg/L h) is defined as the amount of product formation per unit of time. In the present study, we compared rapamycin productivity (d*P/*d*t*) in shake flask with that of bioreactor. Figure 3b represents the difference of rapamycin productivity in shake flask and bioreactor. Comparison of rapamycin production by different strains under various fermentation processes has been described in Table 2. Zhu et al., reported maximum productivity of rapamycin in fed-batch operate mode of 20,000-L fermentor, which is maximum productivity in fermentor reported till date (Zhu et al. 2010). In the present study, maximum rapamycin productivity of 9.13 mg/L h^−1^ was obtained in bioreactor (Table 2).

**Table 2:** Comparison of rapamycin production by different strains under various fermentation processes

Microorganism	Bioreactor	Operate mode	Time (h)	Rapamycin titer (mg/L)	Productivity (mg/L h)	References
*S. hygroscopicus* C9	Shake flask	Batch	144	186	1.29	Fang and Demain (1995)
*S. hygroscopicus* C9	Shake flask	Batch	144	130	0.90	Lee et al. (1997)
*S. hygroscopicus* NBS-9746	130L fermentor	Fed Batch	110	110	1.0	Cheng (1995)
*S. hygroscopicus* N5632	Shake flask	Batch	120	420	3.5	Xu et al. (2005)
*S. hygroscopicusGS*-1437	Shake flask	Batch	120	445	3.71	Chen et al. (2009)
*S. hygroscopicus* R060107	5L fermentor	Fed batch	120	500	4.17	Chen et al. (2008)
*S. hygroscopicus* HD-04-S	20,000L fermentor	Fed batch	168	783	4.66	Zhu et al. (2010)
*S. hygroscopicus* ATCC29253	Tubes	Batch	120	42.8	0.36	Jung et al. (2011)
*S. hygroscopicus* FMT11	7L fermentor	Fed batch	204	860.6	4.22	Zou and Li (2013)
*S. hygroscopicus* MTCC4003	Shake flask	Batch	144	539	3.74	This study
*S. hygroscopicus* MTCC4003	2.2L fermentor	Batch	144	1,316.02	9.13	This study

**Fig. 4:**
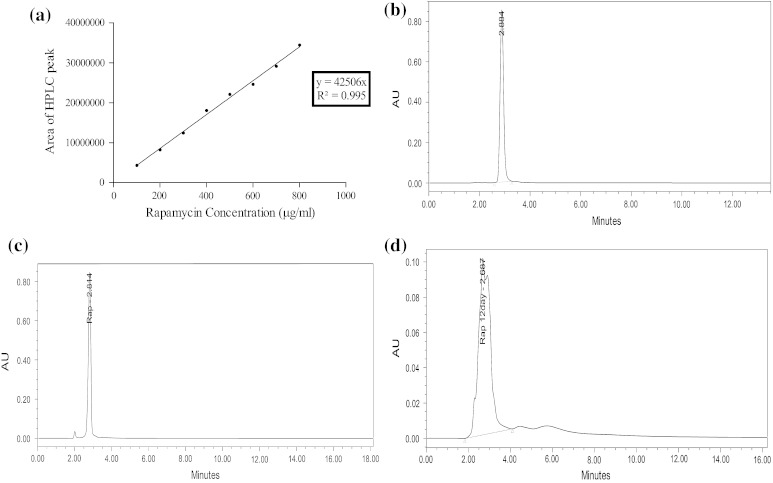
**a** Calibration curve between the HPLC peaks areas and respective concentration of rapamycin standard; **b** HPLC peak of rapamycin standard; **c** HPLC analysis of rapamycin produced on 6th day of fermentation; **d** HPLC analysis of rapamycin on 12th day of fermentation

The production of rapamycin was detected using HPLC analysis. The calibration curve of rapamycin standard was prepared by plotting HPLC peak area against known rapamycin standard concentrations (*R*^2^ = 0.9954) (Fig. 4a). For each concentration of rapamycin standard HPLC retention time (RT) was found to be 2.884 min (Fig. 4b). The methanolic extract of the supernatant was analyzed for presence of rapamycin. Its presence was confirmed by its retention time (2.814 min) which is about same as that of rapamycin standard (Fig. 4c). Figure 3c represents the HPLC chromatogram of methanolic extract of supernatant after 6 days of fermentation. Concentrations of rapamycin in the production medium were obtained by measuring HPLC peak areas (Refaat and Abdel-Fatah 2008). From the equation given in the Fig. 3a, the concentration of the 6th day’s sample was calculated to be 1,316.02 mg/L which is the highest rapamycin produced in the bioreactor till date. It is 52 % higher than that reported previously (860.6 mg/L) by Zou and Li (2013). On the 12th day of fermentation, rapamycin concentration decreased significantly to 91.67 mg/L. This phenomenon was observed by Xu et al. (2005) and might be attributed to the fact that rapamycin starts degrading after 7 days of fermentation (Prapagdee et al. 2008). This is also evident from Fig. 4d that shows the lower rapamycin concentration noted in the 12th day’s sample and formation of two adjacent peaks in the HPLC chromatogram might be attributed to degradation of rapamycin and consequent production of rapamycin degradation products (Prapagdee et al. 2008; Wang et al. 1994).

## Conclusion

Fructose was found to be a better carbon source than mannose. The maximum production of rapamycin in the shake flask was found to be 539 mg/L using fructose as sole carbon source in combination with casein and (NH_4_)_2_SO_4_. Extraction of rapamycin with methanol gave higher yield of rapamycin. Sixth day sample of fermentation gave the highest rapamycin titer of 1,316 mg/L in bioreactor quantified using HPLC technique.

Low titers of rapamycin produced by different strains of *S. hygroscopicus* limits the large-scale industrial production of this potent natural product. Therefore, different attempts, viz. strain improvement, process parameters optimization; precursor engineering studies have been employed to improve the production of this antibiotic. However, proper knowledge of nutritional requirements and kinetic behavior of the organism also need to be thoroughly studied if the maximum production of this immunosuppressant drug is intended. Therefore, the cell growth dynamic results obtained in the present study will help in the enhanced production of this drug.

## Abbreviations

d*P/*d*t*: Production rate (mg/L h^−1^)
*K*_S_: Saturation coefficient (g/L)
*K*_i_: Inhibition coefficient (g/L)
*m*: Cell maintenance coefficient
*P*_0_, *P*: Product concentration at 0th h of fermentation (mg/L), product concentration at particular time of fermentation (mg/L)
*R*^2^: Regression coefficient
*R*_t_: HPLC retention time (min)
*S*_0_, *S*: Initial substrate concentration (g/L), limited substrate concentration (g/L)
*t*: Incubation time (h)
vvm: Volume of air per volume of fermentation media per minute (m^3^)
*X*_0_, *X*: Initial cell mass concentration (g/L), cell mass concentration at any time of fermentation (g/L)
*Y*_X/S_: Growth yield coefficient per unit substrate consumed (g g^−1^)

## Greek symbols

*μ*, *μ*_max_: Specific growth rate (h^−1^), maximum specific growth rate (h^−1^)

## References

[CR1] Bhunia B, Basak B, Bhattacharya P, Dey A (2012). Kinetic studies of alkaline protease from *Bacillus licheniformis* NCIM-2042. J Microbiol Biotechnol.

[CR2] Chen Y, Krol J, Huang W, Cino JP, Vyas R, Mirro R, Vaillancourt B (2008). DCO2 on-line 5 measurement used in rapamycin fed-batch fermentation process. Process Biochem.

[CR3] Chen X, Wei P, Fan L, Yang D, Zhu X, Shen W, Xu Z, Cen P (2009). Generation of high-yield rapamycin-producing strains through protoplasts-related techniques. Appl Microbiol Biotechnol.

[CR4] Cheng YR (1995). Phosphate, ammonium, magnesium and iron nutrition of *Streptomyces hygroscopicus* with respect to rapamycin biosynthesis. J Ind Microbiol Biotechnol.

[CR5] Fang A, Demain A (1995). Exogenous shikimic acid stimulates rapamycin biosynthesis in *Streptomyces hygroscopicus*. Folia Microbiol.

[CR6] Foroncewicz B, Mucha K, Paczek L, Chmura A, Rowinski W (2005). Efficacy of rapamycin in patient with juvenile rheumatoid arthritis. Transpl Int.

[CR7] Graziani EI (2009). Recent advances in the chemistry, biosynthesis and pharmacology of rapamycin analogs. Nat Prod Rep.

[CR8] Jung WS, Yoo YJ, Park JW, Park SR, Han AR, Ban YH, Kim EJ, Kim E, Yoon YJ (2011). A combined approach of classical mutagenesis and rational metabolic engineering improves rapamycin biosynthesis and provides insights into methylmalonyl-CoA precursor supply pathway in *Streptomyces hygroscopicus* ATCC 29253. Appl Microbiol Biotechnol.

[CR9] Kojima I, Cheng YR, Mohan V, Demain AL (1995). Carbon source nutrition of rapamycin biosynthesis in *Streptomyces hygroscopicus*. J Ind Microbiol.

[CR10] Kumar CG, Malik RK, Tiwari MP, Jany KD (1999). Optimal production of *Bacillus* alkaline protease using a cheese whey medium. Microbiologie des Alimentes et Nutr.

[CR11] Lee MS, Kojima I, Demain AL (1997). Effect of nitrogen source on biosynthesis of rapamycin by *Streptomyces hygroscopicus*. J Ind Microbiol Biotechnol.

[CR12] Miller GL (1959). Use of dinitrosalicylic acid reagent for determination of reducing sugar. Anal Chem.

[CR13] Morelon E, Mamzer-Bruneel MF, Peraldi MN, Kreis H (2001). Sirolimus: a new promising immunosuppressive drug. Towards a rationale for its use in renal transplantation. Nephrol Dial Transplant.

[CR14] Nicoletti F, Lapenta C, Donati S, Spada M, Ranazzi A, Cacopardo B, Mangano K, Belardelli F, Perno C, Aquaro S (2009). Inhibition of human immunodeficiency virus (HIV-1) infection in human peripheral blood leucocytes-SCID reconstituted mice by rapamycin. Clin Exp Immunol.

[CR15] Okpokwasili GC, Nweke CO (2005). Microbial growth and substrate utilization kinetics. Afr J Biotechnol.

[CR16] Park SR, Yoo YJ, Ban YH, Yoon YJ (2010). Biosynthesis of rapamycin and its regulation: past achievements and recent progress. J Antibiot (Tokyo).

[CR17] Prakasham RS, Subba Rao C, Sreenivas Rao R, Sarma PN (2007). Enhancement of acid amylase production by an isolated *Aspergillus awamori*. J Appl Microbiol.

[CR18] Prapagdee B, Kuekulvong C, Mongkolsuk S (2008). Antifungal potential of extracellular metabolites produced by *Streptomyces hygroscopicus* against phytopathogenic fungi. Int J Biol Sci.

[CR19] Priest FG (1977). Extracellular enzyme synthesis in the genus *Bacillus*. Bacteriol Rev.

[CR20] Refaat Y, Abdel-Fatah E (2008). Non conventional method for evaluation and optimization of medium components for rapamycin production by *Streptomyces hygroscopicus*. Res J Microbiol.

[CR21] Sabatini DM, Pierchala BA, Barrow RK, Schell MJ, Snyder SH (1995). The rapamycin and FKBP12 target (RAFT) displays phosphatidylinositol 4-kinase activity. J Biol Chem.

[CR22] Sallam L, El-Refai A, Osman M (2010). Some physiological factors affecting rapamycin production by *Streptomyces hygroscopicus* ATCC 29253. J Am Sci.

[CR23] Sanchez L, Brana AF (1996). Cell density influences antibiotic biosynthesis in *Streptomyces clavuligerus*. Microbiol.

[CR24] Schuhmann E, Bergter F (1976). Microscopic studies of *Streptomyces hygroscopicus* growth kinetics. Z Allg Mikrobiol.

[CR25] Sehgal SN (2003). Sirolimus: its discovery, biological properties, and mechanism of action. Transplant Proc.

[CR26] Subba Rao C, Madhavendra SS, Sreenivas Rao R, Hobbs PJ, Prakasham RS (2008). Studies on improving the immobilized bead reusability and alkaline protease production by isolated immobilized *Bacillus circulans* (MTCC 6811) using overall evaluation criteria. Appl Biochem Biotechnol.

[CR27] Tain LS, Mortiboys H, Tao RN, Ziviani E, Bandmann O, Whitworth AJ (2009). Rapamycin activation of 4E-BP prevents parkinsonian dopaminergic neuron loss. Nat Neurosci.

[CR28] Tobisch S, Zuhlke D, Bernhardt J, Stulke J, Hecker M (1999). Role of CcpA in regulation of the central pathways of carbon catabolism in *Bacillus subtilis*. J Bacteriol.

[CR29] Wang CP, Chan KW, Schiksnis RA, Scatina J, Sisenwine SF (1994). High performance liquid chromatographic isolation, spectroscopic characterization, and immunosuppressive activities of two rapamycin degradation products. J Liq Chromatogr Relat Technol.

[CR30] Weber T, Abendroth D, Schelzig H (2005). Rapamycin rescue therapy in patients after kidney transplantation: first clinical experience. Transpl Int.

[CR31] Wiederrecht GJ, Sabers CJ, Brunn GJ, Martin MM, Dumont FJ, Abraham RT (1995). Mechanism of action of rapamycin: new insights into the regulation of G1-phase progression in eukaryotic cells. Prog Cell Cycle Res.

[CR32] Xu Z-N, Shen W-H, Chen X-Y, Lin J-P, Cen P-L (2005). A high throughput method for screening of rapamycin-producing strains of *Streptomyces hygroscopicus* by cultivation in 96-well microtiter plate. Biotechnol Lett.

[CR33] Zhu X, Zhang W, Chen X, Wu H (2010). Generation of high rapamycin producing strain via rational metabolic pathway-based mutagenesis and further titer improvement with fed -batch bioprocess optimization. Biotechnol Bioeng.

[CR34] Zou X, Li J (2013). Precursor engineering and cell physiological regulation for high level rapamycin production by *Streptomyces hygroscopicus*. Ann Microbiol.

